# Exploring the perspectives of young adults on mental healthcare and systemic health, education, and social challenges in Australia: a qualitative study

**DOI:** 10.1186/s12913-025-13580-1

**Published:** 2025-10-22

**Authors:** Zahra Cooper, Bradley Roberts, Georgia Landery, Sarah Woodland, Khan R. L. Collins, Bernadette T. Majda, Susanne Stanley, Anthony Akkari, Sean D. Hood, Jennifer Rodger

**Affiliations:** 1 Perron Institute for Neurological and Translational Science, Nedlands, WA Australia; 2 School of Biological Sciences, The University of Western Australia, Crawley, WA Australia; 3 Rural Clinical School, The University of Western Australia, Perth, 6009 Australia; 4 School of Biomedical Sciences, The University of Western Australia, Crawley, WA Australia; 5 Division of Psychiatry, School of Medicine, The University of Western Australia, Crawley, WA Australia; 6 North Metropolitan Health Service, Western Australian Department of Health, Nedlands, WA Australia; 7 Curtin Medical School, Curtin University, Bentley, WA Australia; 8 Centre for Molecular Medicine and Innovative Therapeutics, Murdoch University, Murdoch, WA Australia; 9 WA Suicide Prevention and Resilience Research Centre (SPARRC), The University of Western Australia, Crawley, WA Australia

**Keywords:** Youth mental health, Mental health service delivery, Access to mental healthcare, Health systems reform, Qualitative research, Systemic challenges to healthcare, Help-seeking behaviour

## Abstract

**Background:**

Young people often face significant challenges accessing effective mental health support as they navigate through complex healthcare systems, education pathways, and social pressures. Understanding the service-level barriers they encounter is critical to improving mental health system design and delivery. While previous studies have examined individual barriers to mental healthcare access, few have adopted a cross-sectorial, youth-informed approach which captures the interrelated structural, institutional, and socio-cultural factors influencing young people’s mental health experiences.

**Methods:**

Seventeen participants aged 18–24 years with lived experience of depression and/or anxiety participated in nine in-person focus groups and interviews in Perth. Reflexive thematic analysis was used to identify systemic barriers and facilitators to mental healthcare, with a particular focus on access, care coordination, therapeutic engagement, and service responsiveness.

**Results:**

Key themes included fragmented care pathways, inadequate provider follow-up, prolonged wait times, financial constraints, lack of youth-specific mental health training among clinicians, and limited therapeutic rapport. Participants also described inadequate mental health literacy within schools and persistent stigma in social and familial settings. These intersecting barriers hindered access, disrupted continuity of care, and undermined trust in the mental health system.

**Conclusions:**

Findings highlight critical inefficiencies in mental health service delivery for young Australians. Policy responses should prioritise integrated care models, investment in multidisciplinary youth mental health hubs, improved school-based mental health literacy, and culturally inclusive anti-stigma initiatives to promote access, trust, and continuity of care.

## Introduction

Youth mental health remains a pressing public health challenge in Australia. Mood disorders, including depression and anxiety, are the most common mental health conditions in young people, often emerging in adolescence and early adulthood and frequently persisting into later life if untreated [1, 2]. Recent data from the Australian Bureau of Statistics indicate that 38.8% of Australians aged 16–24 years reported a mental disorder in the past 12 months, and over a quarter reported high or very high psychological distress [3]. These findings underscore the urgency of ensuring accessible, responsive, and effective mental health services for young Australians.

Mood disorders often persist with significant personal, social, and economic consequences. When experienced in early youth, these disorders can elevate the risk of disease exacerbation, such as an increase in suicidal behaviours, and further impact upon the societal consequences these disorders have on the community [4]. If left untreated, individuals living with a mood disorder are likely to continue to develop chronic symptoms and impairment into their adult years, carrying with them a greater risk of developing a mental health-related disability and placing additional strain on the healthcare system [5]. In Australia, the estimated annual economic cost of mental illness ranges between $200 and $250 billion [6], largely driven by healthcare expenses and lost productivity due to unemployment or reduced capacity to work.

Despite this, young people often perceive the system to be inadequate in addressing their developmental and contextual needs. The division between paediatric and adult services frequently leaves adolescents in a transitional gap, where continuity of care is disrupted [7]. Inadequate integration between primary care, specialist services, and social support systems further compounds these issues, creating barriers to engagement and trust. Poor mental healthcare access during youth can lead to long-term impairments, exacerbation of symptoms, and increased societal costs through lost productivity and health burden [6].

Inadequate incorporation of mental healthcare for youth transitioning from childhood to adulthood results in poor access and care quality [7]. With our current healthcare systems designed with a key focus on paediatric and adult services, adolescents and young adults often fall into a gap and go unnoticed. Understanding how young people engage with the healthcare services that are available for them and their experiences with these systems is essential to develop more effective, youth-centred approaches [8]. Furthermore, addressing systemic barriers impacting healthcare engagement is crucial to improving access, quality, and continuity of care for young people as they transition through these vulnerable stages of mental and emotional development.

A growing body of international research has documented the challenges young people face when navigating mental health systems [9–13], the inadequacies of mental health literacy and shared language [14–16], and the impacts of psychosocial and cultural factors on access and engagement [9, 11, 12]. Collectively, this literature points to persistent structural and implementation-level barriers that undermine early intervention and sustained engagement within youth-oriented services and at the school–health interface [10, 13]. Despite this growing literature, youth-informed research that examines how barriers intersect across healthcare, education and social systems remains scarce. This study addresses that gap by centring the voices of young Australians with lived experience of depression and anxiety and offering an integrated analysis of the barriers they encounter within and between these systems.

This study explores the perspectives of young Australians (aged 18–24 years) with lived experience of depression and/or anxiety, focusing on their interactions with mental health services, schools, and families. The aim is to identify systemic challenges and enablers to care, and to generate evidence-based recommendations for improving service delivery and access within a coordinated, health systems framework.

## Methods

### Participant recruitment

Participants aged 18–24 with self-reported experiences using antidepressant pharmacotherapy were recruited through community organisations, tertiary education institutions, mental health support networks, and social media advertisement. For the purposes of this study, this demographic will be referred to as “youth”, unless otherwise specified. The focus group discussions were audio-recorded and transcribed verbatim to maintain accuracy and reliability in data analysis. Participants were included if they were (1) between the ages of 18–24 years, (2) self-reporting a history of antidepressant use, and (3) were able to converse in English to a proficiency of being able to provide informed consent to participate.

### Data collection

Nine focus groups and interviews were conducted using a semi-structured guide to explore barriers and facilitators to mental healthcare. Focus groups consisted of 2–4 participants; when too few participants were available, interviews were conducted individually. Participants were offered the option to participate either in person or online. However, all sessions were ultimately conducted face-to-face at the Perron Institute for Neurological and Translational Science, located in Perth, Western Australia. This was because data saturation was reached during in-person interviews and logistical factors limited the use of online modalities. Travel expenses were reimbursed to support participation, and while recruitment materials specified the Perth location, there was no requirement for participants to reside in Perth. As a result, the sample included individuals born in various Australian states and internationally, though all were physically present in Perth at the time of interview.

Discussions were guided by a semi-structured framework designed to explore the barriers and facilitators to accessing mental healthcare. Insights from these discussions were captured in vignettes that were woven into the research narrative to enrich understanding of patient experiences. The guiding questions for focus groups and interviews have previously been published [8].

The original objective of these sessions, as part of a larger research project, was to explore barriers and facilitators to accessing and incorporating pharmacogenetic testing in personalising antidepressant medication prescription. During these discussions, participants voluntarily shared broader insights into their interactions with the mental healthcare system, including challenges in accessing and receiving treatment. These concerns suggest that the broader systemic issues within mental healthcare discussed in this study, could impede the adoption of new personalised medicine technologies in mainstream practice. These additional narratives were integrated into the current article’s research narrative to provide a more comprehensive understanding of patient experiences. Thematic saturation was considered reached when new interviews ceased to yield novel themes, a process supported by reflective team discussions.

### Data analysis

This study adopts a systems-based perspective to explore the barriers young Australians face in navigating mental health support. Data analysis was conducted as an inductive, iterative, and reflexively informed process to reveal the nuanced dimensions of participants’ lived experiences. Recognising that researchers’ backgrounds inherently shape the investigative process [17], we continuously reflected on our roles throughout analysis. Initially, two team members (ZC, BR) immersed themselves in the data by repeatedly reviewing transcripts and field notes, developing an intimate familiarity with both explicit content and latent nuances [18]. Salient phrases were systematically annotated during open coding, forming the basis for axial coding that aggregated these codes into broader thematic categories. Throughout, we maintained a deliberate reflexive stance, critically questioning how our positionalities and assumptions might influence coding and interpretation. During the coding process, ZC used selective coding to identify the key themes most relevant to addressing the research question. These codes were further discussed and reviewed with SW. Excel spreadsheets were used for qualitative data organisation and analysis. Themes were developed to inform youth-centred mental health service improvements. Thematic saturation was deemed achieved when successive interviews produced no substantially new codes or categories, as confirmed through iterative team discussions.

Codes and themes were then presented to GL, a research assistant within the age group of participants, and SW, a general practitioner-researcher, to ensure applicability to youth mental health primary care. The diverse expertise of our team further enriched the analysis: ZC’s background in consumer engagement and BR’s direct involvement in focus groups provided valuable firsthand insights, while GL’s shared age cohort with participants contributed a unique insider perspective. This multidisciplinary approach, supported by a broader author team comprising clinicians and academic researchers, enhanced the credibility, depth, and practical significance of our findings, ultimately yielding a richly contextualised understanding of complex human experiences.

### Participant demographics

A total of 32 young people expressed interest in participating in the study. Of these, 17 met the inclusion criteria and participated in either a focus group or an individual interview. Five focus groups were conducted with 2–3 participants each, alongside four one-on-one interviews. The sample comprised predominantly female participants (*n* = 12, 71%), with additional representation from male (*n* = 4, 23%) and non-binary or gender diverse individuals (*n* = 1, 6%). The majority of participants had attained or were pursuing post-secondary education, including those completing an undergraduate degree (*n* = 7, 41%), those who had completed an undergraduate degree (*n* = 3, 18%), and those enrolled in postgraduate study (*n* = 2, 12%). Five participants (29%) had completed high school as their highest level of education. Over half of the participants were born outside Australia (*n* = 9, 53%), and half of the sample for whom data were available (*n* = 8 of 16) reported that both parents were born overseas. Additionally, 41% (*n* = 7) of participants reported speaking a language other than English at home. Overall, the sample reflects a culturally diverse and predominantly tertiary-educated group of young adults.

## Results

### Findings

Thematic analysis of focus group and interview discussions revealed complex perceptions of systemic challenges within healthcare, education, and social systems. Participants expressed their frustration and a sense of disconnection, feeling these systems often failed to address the nuances of their lived experiences. These challenges reflect deep-rooted structural and operational issues that undermine the accessibility, responsiveness, and inclusivity of services designed to support young Australians. This section explores the systemic barriers across these interconnected systems, with thematic categories presented below. Participant voices are used to illustrate underlying issues and emphasise the need for more integrated, empathetic, and youth-centred approaches.

### Healthcare system

Youth participants described various barriers to accessing mental healthcare within the health system. These barriers not only delayed participants from receiving appropriate support but also contributed to feelings of helplessness and frustration in seeking care. Key issues included, fragmentation of care, prolonged wait times, high costs, and a lack of youth-specific expertise among providers resulted in weak therapeutic relationship between patients and providers, and ineffective and inefficient referral pathways.

#### Fragmentation and lack of integrated care

Participants highlighted a systemic lack of integration in the current mental healthcare system, with services often operating in silos that utilise poor mechanisms for patient follow-up. This fragmentation led to young people feeling as though they had to repeatedly retell their stories to different healthcare providers, an emotionally taxing process that eroded trust and delayed treatment interventions. Participants suggested solutions such as implementing electronic health records to improve communication and continuity of care. For example.

*I’ve had a really bad experience where I was like bounced back and forth from clinician to clinician. And just the reiterations of having to go through the different professionals and having to restart my file again*,* it was quite exhausting. Um*,* so then I actually stopped seeking professional help for a while and then it kind of got to another crisis point again. [FG2P2]*

Referral processes were often described as confusing and inefficient. GPs, who were often seen as being unsure how to manage complex mental health cases, tended to “*brush aside*” patients, referring them to other professionals without clear guidance or follow-up.

*So*,* I found it was hard to find like a suitable psychologist or psychiatrist because the GP didn’t really have any connections professionally so they weren’t really able to recommend anyone I guess that would maybe be more suitable […]* [FG7P1]

*GPs might just not know what to do with that thing…like ‘I’m not the one to handle this. You can go somewhere else’.* [FG2P2]

These inefficiencies in referral pathways contributed to delays in receiving appropriate care and added to the overall frustration experienced by participants.

#### Prolonged wait times

Delays in care access was one of the most frequently cited issues. Youth participants reported waiting weeks or months for appointments, with wait times for specialists such as psychiatrists or counsellors, often exacerbating their mental health struggles. This lack of timely intervention created feelings of abandonment and discouraged further help-seeking behaviour.

*[…] it took me a really long time to*,* to get another appointment at the psychiatrist cause there was another wait time.* [FG2P2]

*There’s such a huge wait to like*,* even be able to see [a psychiatrist] and I feel like this like a sort of stress.* [FG7P2]

This barrier is especially concerning for young people experiencing acute mental health issues, as prolonged delays can exacerbate symptoms and increase the risk of crisis events from presenting. The perception that mental health services are inaccessible when needed discourages youth from pursuing help, reinforcing a cycle of unmet mental health needs.

#### Cost of specialised mental healthcare

The financial burden of accessing mental healthcare, particularly for secondary and tertiary students, as well as recently independent youth who may be on a lower income, emerged as a significant barrier to getting sufficient care. Participants described being unable to afford ongoing care, medications, or therapy due to the financial burden.

*I stopped seeing professionals because it was getting too expensive.* [FG7P1]

The inadequacy of government support and private insurance in covering mental health costs further compounded this issue, with participants expressing frustration at the financial strain of seeking care.

*Without private health insurance*,* which doesn’t cover a lot*,* and being a [University] student*,* I can’t really afford to keep seeing them regularly.* [FG7P1]

#### Knowledge gaps in youth-specific mental health

Participants noted that they often felt that providers lacked an understanding of the unique challenges and stressors faced by young people, and that healthcare providers lacked adequate preparation to address complex mental health issues among youths. Many felt that healthcare providers, particularly in the primary care system, were unprepared for the depth and breadth of their needs and lacked the nuanced knowledge necessary to address the unique developmental, social, and emotional burdens faced by young people, leading to a superficial or inadequate treatment approach.

*[…] only like recently after like 4 years*,* they gave me something good. So*,* a lot of time wasted trying to find the right*,* yeah. […] I also find them very like under…prepared.* [FG2P1]

Participants also noted that some providers appeared unsure about how to manage younger patients effectively.

*[…] from what I could recall*,* I think at that time they weren’t really sure what to do? They didn’t know how to support me because I think at that time*,* I was underage*,* and I think they weren’t sure about […].* [FG2P2]

#### Limited therapeutic relationship

Many participants reported feeling dismissed or undervalued by their GPs, citing a lack of empathy and inadequate communication. These negative experiences diminished their trust in their GP’s guidance and treatment plan and deterred them from seeking further help.

*I’ve only ever met one GP who seemed to care what happened to me.* [FG2P3]

In another case, a participant recounted feeling as though their mental health concerns were trivialised:

*When I went to my GP in Year 12*,* she was really dismissive of my mental health and made me feel like I was exaggerating.* [FG7P2]

This participant believed that the GP dismissed their mental health struggles by suggesting they were exaggerating or influenced by what they saw in the media. The participant felt invalidated and “*shocked*”, explaining that this experience made them feel “*so stupid*” and discouraged them from returning to his GP or seeking help from another.

These narratives suggest that a perceived lack of empathy can lead to disengagement, particularly in the context of mental healthcare, where trust and rapport are foundational to effective treatment.

Another recurrent problem in this study was participant frustration with the quality of communication received from GPs. Participants often reported feeling inadequately informed about their diagnoses, treatment options, and the potential side effects of medications. Though many participants understood the brevity of consultations, this lack of comprehensive communication contributed to feelings of disempowerment and dissatisfaction with the care they received.

*I think*,* like obviously there’s time restraints in GP appointments*,* but a bit more of a conversation*,* not just like ‘here you go*,* see ya. Try it out. Good luck.’* [FG3P2]

Another participant stated that their GP failed to explain the potential side effects of their medication, leaving them to return later with a constant headache. They noted that they would have preferred to be informed upfront, which would have helped manage their expectations and build trust in their GP’s care.

*I don’t know why they don’t tell you the side effects beforehand. Especially when it’s your first time trying a new thing.* [FG7P3]

One participant described a lack of adequate discussion regarding their mental health. After being prescribed medication, they were given printouts about their condition but felt this method was inadequate for truly understanding their treatment. They indicated that more conversation was needed, rather than just “*pages and pages*” [FG3P2] of printed information.

Despite the overall trend of dissatisfaction, several participants recounted positive experiences with GPs who provided attentive and empathetic care. These accounts serve as important counterpoints, illustrating the potential for GPs to foster trust and improve patient experiences through thoughtful engagement. For example, one participant highlighted the benefit of the continuity of care that their GP provided:

*In fairness to her*,* she did make me book follow-up appointments so she could check in on me.* [FG1P1]

Another participant appreciated the comprehensive approach their GP took:

*I saw another GP in my first year of university*,* and she was less dismissive. She suggested a blood test to check if it was something like low iron or another issue.* [FG7P2]

These examples suggest that limited communication within time-pressured appointments can significantly diminish the quality of care and leave patients feeling unsupported during their treatment journey. However, the positive experiences underscore the significant impact that thorough consultations and follow-up care can have on young people’s perceptions of their care, particularly when dealing with sensitive mental health concerns. This is particularly important to youth accessing support for mental health issues.

### Education system

The education system plays a pivotal role in shaping youth experiences with mental health, with 14 of our 17 participants starting their mental health journey during high school years. However, participants identified significant shortcomings, including inadequate mental health literacy among educators, insufficient resources, and pervasive stigma within schools. These gaps often left students feeling unsupported and isolated, particularly during periods of heightened stress.

#### Mental health awareness and training

Many participants expressed concerns that the high school environment lacks adequate mental health literacy and that teachers are often underprepared to address student mental health needs. This gap results in young people feeling misunderstood or unsupported. Participants express a strong desire for teachers to receive mental health training to improve awareness and support for students facing emotional challenges.

*[…] for schools to have more information. Whether that’s like actual [classes] they do on mental health or whether it’s just teachers having better understanding so they can spot signs and symptoms.* [FG4P1]

This participant also recommended incorporating mental health literacy into school curricula to normalise discussions around emotional wellbeing and equip both students and staff with the tools to recognise and respond to challenges.

*You learn about physical health and yeah*,* the at least when I was in school*,* there was nothing on mental health. Yes. So that needs to change.* [FG4P1]

*[…] it can be quite a difficult thing to ask for help and to seek information. So having like information like even pamphlets or whatever like more available. […] Especially for kids who literally cannot*,* there is no other way for them to get help*,* […] education*,* even from like*,* forced education*,* like from a teacher*,* classes*,* like pamphlets given out to each kid*,* yeah…* [FG1P1]

*[…] school is like for a lot of kids*,* it’s the only safe space. Yeah*,* and having mental health resources*,* there is incredibly important.* [FG6P1]

Participants also highlighted the variability in teacher engagement, with some educators taking proactive steps to support students, while others appeared indifferent or unequipped to help.

*I had like one teacher who I just had a good relationship with*,* so she was but the school in general*,* I don’t think really was. I saw the school counsellor like twice and then she kind of*,* you know*,* referred me to my*,* back to my GP and then to other services. And so kind of when it went outside of the school*,* the school kind of lost interest or forgot…* [FG4P1]

Participants also mentioned the lack of trained mental health professionals within schools. Participants reported that many schools relied on underqualified staff to manage mental health services, reflecting broader underfunding and resource constraints.

*My school had someone random’s mum do an online course on youth mental health…but she was also the admin assistant.* [FG1P1]

Some participants stated a lack of awareness around available resources as a barrier to students seeking or accessing help for their mental health challenges.

*[…] not even the school psychologists cause I didn’t know if they had. I don’t know why I didn’t know if they had a school psychologist.* [FG2P2]

This theme reflects a broader issue of awareness and insufficient mental health resources in educational and community settings, with significant gaps in support for young people.

#### Stigma and peer-related issues within the school environment

Mental health stigma in schools discouraged many participants from seeking help. They noted that stigma often led to feelings of shame and isolation and reinforced a culture of silence around mental health struggles.

*Yeah*,* that it was like ‘ohh*,* you know you’re that kid.’ So it was very like hush hush kinda like*,* why you getting pulled out of class all the time then you just like*,* doesn’t nothing that’s going on.* [FG6P1]

This stigma was particularly pronounced in smaller communities, where mental health struggles were often dismissed or ridiculed.

*Um a lot of the help I got was external out of school because it was just like kind of separated. Um*,* but especially where I went to school*,* I went to school in like small town*,* middle of nowhere-Western Australia. So*,* there was very much still the stigma around mental health.* [FG6P1]

These findings highlight the critical need for systemic reforms in education to address mental health challenges comprehensively. By improving teacher training, increasing access to resources, and actively combating stigma, schools can create more inclusive and supportive environments for young people.

### Societal system

Some participants described a persistent stigma within their social networks and families, significantly hindering their willingness to seek help or discuss mental health challenges. This section explores the dual impact of societal stigma and family attitudes on youth mental health, illustrating the urgent need for cultural and systemic changes to foster supportive environments.

#### Stigma and social support

Stigma around mental health remains pervasive within social networks, causing youth to hesitate in seeking help or discussing their struggles with peers and family. Participants report feeling judged or misunderstood when they try to open up, leading to isolation and reinforcing negative perceptions about mental health. This stigma acts as a barrier to accessing available support and sharing their experiences.

*when I was in high school*,* […] I was forced to go see a youth psychiatrist or whatever*,* um*,* but the stigma surrounded it*,* not only for my family*,* um*,* which I couldn’t get any medications or anything at that time cause my family would have said no*,* um*,* but also the school I went to*,* the community that I was in. […] Then when I finally had the capacity to go myself that stigma remained and I couldn’t quite uh*,* like get the proper help I needed.* [FG1P1]

For some, stigma extended beyond their immediate social networks to encompass broader cultural attitudes. Participants from culturally diverse backgrounds often faced compounded challenges due to entrenched negative views on mental health.

*[…] growing up in an obviously Asian family*,* I had a lot of stigma*,* um around mental health*,* um*,* challenges and*,* and*,* um illness*,* in general […].* [FG2P2]

*I think […] the country where I come from*,* there’s so much of negativity around the term [mental health disorders].* [FG4P2]

Family attitudes and support: Family resistance to mental healthcare emerged as a critical barrier for dependents under the age of 18 years, with participants describing opposition to therapy and medication. This resistance often stemmed from misconceptions, lack of awareness, or cultural beliefs, leaving many young people to navigate their mental health journeys alone.

*[…] both my parents were really against anything to do with mental health […]. So*,* Mum didn’t want me to like see anyone […] and Dad was even worse than that.* [FG2P3]

*[…] I couldn’t get any medications or anything at that time cause my family would have said no.* [FG1P1]

For those recounting times when they were underage, parental resistance created additional challenges, as consent was required to access professional help. Participants emphasised the importance of increasing family awareness and education to foster more supportive home environments.

*[…] because you’re still a minor and without parental support to go through […]. It wasn’t until I was 18*,* an adult*,* and that’s when I took over my own health because there wasn’t really support.* [FG7P1]

Privacy concerns were also highlighted, with some participants expressing the need for discreet ways to access care without family interference or judgment.

[interviewer: And were your parents…[aware]? ] *They didn’t know. They still don’t.* [FG4P2].

Despite these barriers, a few participants described moments when family involvement eventually became a turning point, albeit late in their mental healthcare journey.

*Eventually my parents had to bring me to go see a*,* like*,* seek professional help.* [FG7P3]

These narratives underscore the critical role of social and familial environments in shaping mental health experiences. Addressing stigma and fostering understanding within these spheres is essential to breaking down barriers and supporting young people in their mental health journeys.

## Discussion

This study highlights significant inefficiencies and inequities in mental health service delivery for young Australians. It provides insights into the systemic barriers shaping the experiences of young adults with lived experiences of depression and/or anxiety within healthcare, education, and societal systems. It’s important to note that while this study provides important insights into young people’s experiences of the Australian mental healthcare, all data were collected in-person at the Perron Institute in Perth, Western Australia. While participants varied in place of birth, including several born outside Australia, all were physically present in Perth at the time of data collection. The study’s findings reflect the experiences of young people in this metropolitan context and may not reflect the perspectives of those living in regional, rural, or interstate contexts.

Findings reveal intersecting barriers across all three systems, with significant gaps in health service provision, fragmented care pathways, insufficient mental health literacy among educators, and the pervasive stigma surrounding mental health. These barriers are found to undermine access and continuity of care. At the heart of these insights is a youth-informed critique of existing systems; a recognition that the lived experiences of young people are not merely anecdotal but form a critical evidence base for shaping future reforms, echoing calls in recent reviews to centre youth and family perspectives when redesigning services across sectors [9–11, 13]. The following discussion expands upon these barriers and offers research-backed, policy-relevant solutions.

### Healthcare system

The fragmented nature of Australia’s mental healthcare system emerged as a primary obstacle for youth seeking care. A lack of continuity and inadequate follow-up mechanisms meant that participants often had to repeatedly recount their mental health histories to multiple providers. These findings align with findings reported in both youth and older adults [19, 20], and across various healthcare settings [21, 22]. In particular, studies examining the transition from child to adult mental health services have consistently shown that poorly planned or abrupt transitions contribute to disengagement, interrupted therapeutic relationships, and significant emotional burden for young people [23–25]. Implementing integrated care models, such as shared electronic health records, could enhance communication and coordination, reducing this burden.

Delays in accessing mental healthcare exacerbated by prolonged wait times and high costs further compound these challenges. These findings are consistent with evidence of the strain on Australia’s mental health infrastructure [26]. These experiences echo systemic concerns raised in the Productivity Commission’s Interim Report on the Mental Health and Suicide Prevention Agreement [27], which identifies fragmented care, poor coordination, and disjointed transitions between services as key failures in the current system. Addressing structural inequities – particularly inadequate funding and workforce shortages – through targeted investments, streamlined referral processes, and expanded subsidised services could significantly improve outcomes.

Engagement and the therapeutic alliance are important for young people seeking mental health support, particularly as they may often feel emotionally overwhelmed or uncertain about treatment. Participants described varied experiences, highlighting the importance of empathy, effective communication, and consistent care. Literature supports that positive therapeutic alliances are linked to better outcomes, especially when providers are attuned to youth-specific developmental needs [28]. Improving therapeutic engagement, particularly in primary care, and creating integrated care models could help address concerns raised by participants. This approach is consistent with evidence suggesting that early intervention built upon trusting relationships may reduce long-term mental health difficulties [29].

These improvements must be considered in the context of a chronically underfunded Medicare system in Australia, which places significant time pressure on GPs [15, 16], limits rebates for longer consultations, and increases out-of-pocket costs for patients [30–32]. Younger people are particularly disadvantaged because they have fewer financial resources and are paying substantially more out-of-pocket than older age groups [33]. Similar funding constraints affect access to psychiatrists, with higher gap fees and shortages of specialist providers contributing to longer wait times [33–35]. Psychological treatment delivered by clinical psychologists, general psychologists, and mental-health accredited occupational therapists and social workers through the Better Access initiative also comes with increasing out-of-pocket expense, and is restricted to ten Medicare-rebated sessions per calendar year [31]. This contributes to inequity of access and undertreatment particularly among youth with more complex needs [32]. Without concurrent investment in structural reforms to the Medicare funding model, aspirations for better therapeutic engagement may not be fully realised.

In addition, late adolescents frequently face challenges transitioning from paediatric to adult healthcare, often falling into a service gap, too old for paediatric services yet not fully accommodated by adult care [36, 37]. The Western Australia Department of Health’s Clinical Senate report emphasises the need for structured transition processes to ensure continuity of care for young people moving between service systems [36]. This systemic fragmentation is also present within the tertiary hospital system. This underscores broader calls for dedicated adolescent and young adult mental health infrastructure, including integrated youth hubs in primary care [7].

### Education system

Schools serve as a critical point of contact for youth mental health intervention. However, this study reveals that many educators lack the mental health literacy necessary to identify and support struggling students, leaving young people without adequate support during formative years when early intervention is crucial. National initiatives such as *Beyond Blue’s Be You* framework *aim to increase mental health literacy*,* reduce stigma*,* and enhance early intervention*,* by supporting educators in promoting mental health and wellbeing in Australian schools* [38]. *Such initiatives* represent steps towards embedding wellbeing in school environments. Yet, participants in this study suggest further reform is needed to ensure consistent, qualified, and stigma-free care.

Embedding mental health education in school curricula has been shown to improve mental health literacy and reduce stigma among both students and educators [39], which may not only equip students with the language and tools to understand and articulate their mental health experiences but also foster environments where help-seeking is normalised [40, 41].

Equally important is ensuring adequate access to appropriately trained on-campus mental health professionals. Depending primarily on staff without specialised mental health expertise may delay early identification and intervention for students. Teachers frequently report feeling underprepared to effectively support student mental health needs [42], highlighting the need for accessible, embedded professional support. Students similarly perceive variability in how wellbeing is addressed within school contexts, further indicating opportunities for improved consistency in support practices [42].

Evidence from a recently published systematic review also indicates that targeted professional development for teachers can significantly improve their knowledge, reduce stigma, and enhance referral practices [43]. Such initiatives could support schools in becoming environments that prioritise students’ emotional wellbeing alongside academic learning. These approaches also align with recommendations from the Productivity Commission’s Interim Report on the Mental Health and Suicide Prevention Agreement [27], which calls for stronger community-based mental health services and preventive supports tailored to educational contexts.

### Societal system

The pervasive stigma surrounding mental health remains a significant barrier, particularly within familial and social contexts. Addressing these challenges requires tailored interventions that engage not only young people but also their families and communities, fostering collective accountability in destigmatising mental health. Emerging research highlights the protective role of peer support networks and youth-led mental health initiatives. Programmes that position young people as mental health ambassadors or peer educators have shown promise in reducing stigma, promoting help-seeking, and creating safer social environments for disclosure [44]. Scaling up these youth-led models – particularly in schools, universities, and online platforms – may foster supportive peer cultures and reduce the social barriers that inhibit early intervention.

Participants in this study from culturally diverse backgrounds reported compounded challenges due to entrenched cultural beliefs and resistance to mental healthcare. These findings reinforce the need for targeted anti-stigma campaigns and culturally sensitive approaches, as research has shown that culturally diverse communities often hold distinct beliefs about mental illness that can hinder open discussion and help-seeking [45]. In particular, the interplay between public stigma and self-stigma can significantly erode individual agency, especially among marginalised youth [46]. Addressing these dynamics requires tailored strategies that not only respect cultural values but also actively dismantle barriers to mental health literacy, trust, and access to care.

Community-driven education and dialogue, facilitated by peers, elders, and mental health professionals, can help reframe these narratives. Programs like *Time to Change* in the United Kingdom demonstrate that sustained, targeted campaigns are effective in reducing stigma and increasing mental health literacy across age and cultural groups [47]. In Australia, adapting such initiatives to suit multicultural contexts could empower youth to seek support while simultaneously shifting community norms towards greater acceptance and understanding. These efforts could also be extended to digital platforms, where young people frequently engage in mental health discourse. Social media and digital platforms increasingly function as informal mental health ecosystems, offering spaces for expression, connection, and sometimes misinformation. While these platforms can help reduce isolation, they may also perpetuate stigma or unrealistic norms [20]. Future initiatives should consider how to leverage these digital spaces to promote accurate, inclusive mental health messaging and foster supportive online communities, particularly among culturally diverse youth who may be more active online due to barriers in traditional help-seeking avenues.

### Overlaps and interdependencies of the three systems

A key takeaway from this study is that these systems do not operate in isolation. For instance, a lack of mental health support in schools often places greater pressure on the healthcare system, while stigma within social settings can deter engagement with both educational and healthcare resources. These overlapping barriers require coordinated interventions that integrate mental health literacy, reduce stigma, and ensure continuity of care across systems.

### Policy and practice implications

This study highlights how barriers across healthcare, education, and societal systems are deeply interconnected. Cross-sectoral reforms are necessary, as education and social stigma barriers further exacerbate healthcare service inefficiencies. For example, partnerships between schools and healthcare providers can streamline referrals, while community programs can work to destigmatise mental health and promote awareness.

Policy reforms must prioritise implementing integrated youth mental health hubs with multidisciplinary teams. Models such as *headspace*, under the National Youth Mental Health Foundation, provide a scalable template for integrated, early-intervention care tailored to young people’s developmental needs. Embedding shared electronic health records across primary and secondary mental healthcare can address the system fragmentation experienced by youth. Furthermore, expanding bulk-billed services and subsidising youth-specific mental health services is critical to improving affordability and accessibility [48].

In addition to integrated service hubs, the use of care navigation roles or peer-based support may help guide young people through complex service pathways, particularly during referral transitions. While not without implementation challenges, these supports could help reduce disengagement by providing continuity and clarity in service access [7].

Improving mental health literacy training for educators and the inclusion of mental health literacy in school have shown promise in normalising discussions around emotional wellbeing [39]. Additionally, the presence of qualified mental health professionals within schools may improve early intervention strategies. By integrating mental health education and awareness into the curriculum, schools can address stigma and promote resilience among students.

Perceived stigma is repeatedly identified in reviews as one of the top reasons young people defer or decline services; anti‑stigma strategies that pair literacy with visible youth‑friendly access points appear most promising, albeit with modest effect sizes to date [48, 49]. Community-based anti-stigma initiatives, tailored to reflect the values and norms of diverse populations, are essential for fostering more inclusive and supportive environments. These efforts help to shift public attitudes, challenge misconceptions, and promote understanding. Family education is also a critical component in reducing stigma. When families are equipped with accurate knowledge about mental health, they are more likely to recognise early signs of distress, respond with empathy, and support help-seeking behaviours [45]. Strengthening mental health literacy at the community and family level can thus play a transformative role in breaking down stigma and encouraging timely, appropriate support.

## Limitations and future research

While this study offers valuable insights, several limitations must be acknowledged. The relatively small and purposefully selected sample may limit the generalisability of the findings. However, the research’s intent was to explore participants’ lived experiences deeply rather than to generalise. Reliance on self-reported experiences may introduce biases related to recall and personal interpretation, but these perspectives are critical for capturing the nuanced realities of mental healthcare access.

The focus on young adults aged 18–24 means that the study does not capture the experiences of adolescents under 18, who may face distinct challenges in accessing mental healthcare. Including younger age groups in future research could help identify age-specific barriers and inform more targeted interventions.

The study’s sample was primarily tertiary-educated and limited to participants who could attend in-person sessions in Perth metropolitan region. While these participants offered rich insights into service navigation and structural barriers, findings may not be fully representative of all young Australians, particularly those from socioeconomically disadvantaged or geographically remote communities. Cultural influences on mental health service access warrant further investigation in more diverse cohorts. Future research should aim to incorporate more diverse samples to capture a broader range of perspectives. Heterogenic samples of youth with lived experiences of mental health disorders along with their parents, care and mental healthcare practitioners may also enrich the data and provide more nuanced perspectives. Moreover, further exploration into culturally specific mental health challenges and solutions could provide targeted insights for addressing stigma and resistance in diverse communities. Additionally, exploring the interactions between healthcare, education, and societal systems at a structural level could provide deeper insights into designing coordinated interventions. The role of emerging technologies, such as telehealth and digital mental health tools, should also be explored to bridge gaps across these interconnected systems.

## Conclusion

This study highlights systemic barriers faced by young Australians and offers directions for reform across healthcare, education, and societal systems. The findings underscore the urgent need for reforms that prioritise integration, accessibility, and inclusivity across these sectors. By addressing fragmentation in healthcare, enhancing mental health literacy in education, and challenging stigma through community and family initiatives, policymakers and practitioners can create environments that better support young people’s mental health and wellbeing. The voices of youth are indispensable in shaping effective and responsive solutions. By centring their experiences in policy and practice, stakeholders can ensure that systemic reforms meet the unique needs of this demographic. Ultimately, the adoption of youth-centred approaches will not only improve mental health outcomes but also contribute to a more equitable and empathetic society.

## Data Availability

No/Not applicable (this manuscript does not report data generation or analysis).
